# “Just the way we always did it”: ophthalmologist perspectives on changing routine anesthesia care for cataract surgery in the United States

**DOI:** 10.1186/s13741-026-00697-y

**Published:** 2026-05-11

**Authors:** Nora Lyang, Rachel Schwartz, Saras Ramanathan, Neeti Parikh, Meghan Lane-Fall, Daniel Dohan, Catherine L. Chen

**Affiliations:** 1 Western University of Health Sciences, College of Osteopathic Medicine of the Pacific, Pomona, CA USA; 2 Department of Anesthesia & Perioperative Care, University of California San Francisco, 521 Parnassus Avenue, San Francisco, CA USA; 3 Division of General Internal Medicine, Department of Medicine, University of California San Francisco, San Francisco, CA USA; 4 Department of Ophthalmology, University of California San Francisco, San Francisco, CA USA; 5 Vagelos College of Physicians and Surgeons, Columbia University, New York, NY USA; 6 Philip R. Lee Institute for Health Policy Studies at UCSF, San Francisco, CA USA; 7 Center for Health Equity in Surgery and Anesthesia, University of California, San Francisco, San Francisco, United States

**Keywords:** Cataract surgery sedation, Qualitative study, Health services research, Quality of care, Anesthesia, Anesthesiology, Patient safety, Cataract surgery, Ophthalmology, Health care value

## Abstract

**Background:**

Cataract surgery is one of the most common elective outpatient surgeries performed among older adults in the United States. Modern surgical technique makes cataract surgery a relatively quick, low-risk, minimally invasive outpatient procedure that can be performed solely under ophthalmologist-directed local or topical anesthesia. However, in the US, the procedure is routinely performed with additional monitoring and intravenous sedation administered by anesthesia-trained personnel. Given the procedure’s safety profile, we sought to characterize ophthalmologists’ perspectives on barriers preventing the adoption of more individualized approaches to cataract surgery sedation that do not automatically default to employing routine anesthesia care.

**Methods:**

This study was conducted between December 2022 to January 2024. Using a semi-structured interview guide developed with the Consolidated Framework for Implementation Research (CFIR) framework, we completed interviews with ophthalmologists who performed cataract surgery with or without anesthesia care in hospital outpatient departments, ambulatory surgery centers, minor procedure rooms, and/or office-based surgery suites across the United States. Data were analyzed using an inductive thematic analysis approach to uncover descriptive themes.

**Results:**

We interviewed 19 ophthalmologists (5 women), including 6 who had experience routinely performing cataract surgery without anesthesia care. Three major themes emerged: (1) inertia, defined as the tendency to continue established practices that serve to maintain the status quo, (2) financial considerations, and (3) the interdependent relationship between ophthalmology and anesthesiology. While some participants supported a more selective approach to the use of anesthesia services, study participants identified multiple barriers to change, including difficulty overcoming the current inertia, decreased reimbursement for office-based surgery, and concerns about implications for patient safety without routine anesthesia involvement.

**Conclusion:**

Ophthalmologists identified multiple barriers to changing current anesthesia-led sedation models for cataract surgery. Our findings can help inform future efforts to better align anesthesia care with patient and procedural needs in an aging US population.

**Supplementary Information:**

The online version contains supplementary material available at 10.1186/s13741-026-00697-y.

## Introduction

Cataract surgery is a commonly performed elective surgical procedure among older adults (Schein et al. 2000; Sharwood et al. 2008; Perumal et al. 2022; Navaleza et al. 2006). Modern surgical technique makes cataract surgery a relatively quick, low-risk, minimally invasive, outpatient procedure that can be performed solely under ophthalmologist-directed local or topical anesthesia (Sharwood et al. 2008; Gogate et al. 2005). However, in the US, the procedure is routinely performed with additional monitoring and intravenous sedation administered by anesthesia-trained personnel. This practice is in contrast with the “opt-in” approach to requesting anesthesia care during other types of low-risk outpatient procedures such as cardiac catheterization and upper and lower gastrointestinal endoscopy, where anesthesia care is typically allocated according to patient-specific characteristics that might increase the risk of sedation without additional anesthesia support (Perumal et al. 2022). Additionally, cataract surgeries performed in other countries do not rely as heavily on routine anesthesia care (Chung et al. 2019; Eke and Thompson 2007; Eichel and Goldberg 2005), and some US ophthalmologists have started performing cataract surgery without routine anesthesia care in office based surgery suites (Kugler et al. 2023; Ianchulev et al. 2016).

Given the procedure’s overall safety profile, it is unclear why the same approach to risk stratifying anesthesia care has not yet permeated cataract surgery sedation practice in the United States. Our prior work has shown that ophthalmologists acknowledge the low-risk profile of cataract surgery (Lyang et al. 2025). While ophthalmologists generally agreed that many patients could likely undergo cataract surgery without routine anesthesia care, many expressed discomfort with the idea of giving up the safety net provided by licensed anesthesia providers, especially in the face of unanticipated systemic medical events in a high-risk older adult population (Lyang et al. 2025). Furthermore, there may be additional barriers preventing ophthalmologists from changing their approach to cataract surgery sedation unrelated to their personal perception of risk.

To better understand ophthalmologists’ perspectives on the barriers to implementing cataract surgery sedation approaches that do not require routine anesthesia care, we conducted semi-structured interviews with ophthalmologists who routinely performed cataract surgery in the United States. The goal of this study was to characterize physician perspectives on potentially adopting a more individualized approach to allocating anesthesia care for patients undergoing cataract surgery.

## Methods

This study was approved by the Institutional Review Board at the University of California, San Francisco (IRB# 21—35606). Patient eligibility and data collection followed the Consolidated Criteria for Reporting Qualitative Research (COREQ) guidelines and content analysis methodology (Tong et al. 2007; Krippendorff 2018). A detailed description of participant recruitment, data collection, and data analysis procedures was previously published in this journal (Lyang et al. 2025), which we describe briefly below.

### Participant eligibility, recruitment and data collection

The research team used purposive sampling to recruit US ophthalmologists in diverse clinical and geographic locations who have routinely performed cataract surgeries in their clinical practice (Lyang et al. 2025). Consent was obtained via DocuSign and verbally reaffirmed prior to each interview, and all participants completed a REDCap pre-interview survey capturing demographics and practice characteristics. Using the Consolidated Framework for Implementation Research (CFIR) (Damschroder et al. 2022), a semi-structured interview guide was developed, pilot tested and refined prior to data collection. The study PI (CLC) conducted all interviews virtually with study staff present for notetaking, and all interviews were recorded and transcribed. Upon interview completion, participants received a $200 electronic gift card.

### Data analysis

Data were analyzed following best practices for inductive thematic analysis (Braun and Clarke 2006). The coding team (CLC, NL, RS) used Atlas.ti software (Version 23.3.0) to iteratively define and develop a codebook that reflected the data’s most salient themes. After establishing intercoder reliability, a full 20% of the data were coded by at least two coders and reviewed for internal consistency and rigor, with the first author coding the remaining transcripts. Any discrepancies were resolved through discussion by the coding team until consensus was achieved.

Weekly case summaries of each interview allowed for members of the coding team to transparently reflect and re-evaluate personal biases to maintain reflexivity. The coding team’s different professional backgrounds included (CLC): a female practicing anesthesiologist and senior author with a background in health services research, (NL): a female clinical research coordinator completing a medical degree, and (RS): a female implementation scientist and health services researcher with qualitative expertise. Including team members both within and outside of medicine, and across different career stages, allowed for diverse perspective integration throughout data collection and analysis.

## Results

Among the 19 participants, 8 (42%) currently practiced in academic settings, 14 (74%) were men, and 12 (63%) had 15 years or less of clinical practice experience. Six (32%) participants currently performed cataract surgery without routine anesthesia care (aka “non-MAC”). Among the 13 (68%) participants whose current sedation approach still employed routine anesthesia care, 8 had prior non-MAC experience. Participant demographics can be found in Table 1.

**Table 1:** Self-reported study participant demographics

Total Ophthalmologists (*N* = 19)	**N (%)**
Gender	
Male	14 (73.7%)
Female	5 (26.3%)
Age	
< 35	0 (0%)
36—45	9 (47.4%)
46—55	5 (26.3%)
56—65	3 (15.8%)
66 +	2 (10.5%)
Practice Setting^1^	
Academic	8 (42.1%)
Private Practice	14 (73.7%)
Other	2 (10.5%)
Current Sedation Approach	
MAC	13 (68.4%)
Non-MAC	6 (31.6%)
Practice Duration	
0—5 Years	1 (5.3%)
6—10 Years	7 (36.8%)
11—15 Years	4 (21.1%)
16—20 Years	2 (10.5%)
21 + Years	5 (26.3%)
Current Practice Region	
West	9 (47.4%)
Midwest	2 (10.5%)
South	7 (36.8%)
Northeast	1 (5.3%)
Yearly Cataract Volume	
0—50	0 (0%)
51—100	1 (5.3%)
101—200	3 (15.8%)
201—300	6 (31.6%)
301—400	2 (10.5%)
401—500	2 (10.5%)
501—1000	4 (21.1%)
> 1000	1 (5.3%)

^1^Does not add up to 100% because some physicians practice in multiple settings

While the notion of thematic saturation is increasingly being challenged in the literature (Thorne 2020; Saunders et al. 2018) we noticed no new themes emerging after 17 interviews. The study team then conducted 2 more interviews to confirm that no new themes emerged before data collection stopped and was considered complete. Interview length ranged from 31 to 72 min (median = 52 min, IQR = 16 min). Interview content was synthesized into three major themes focused on 1) inertia, 2) financial considerations, and 3) the interdependent relationship between ophthalmology and anesthesiology, which were each further divided into three subthemes. Additional illustrative quotes and the interview guide can be found in Tables 2, 3, 4, and Supplemental Fig. 1, respectively.

**Table 2:** Additional illustrative quotes regarding theme 1: inertia

Inertia	
*Lack of Evidence*	***O3**** I think it’s helpful from an evidence-based standpoint to have enough publications about comparable safety patient experience to having A versus B, and if it’s comparable, then I think ****it’s always easier to have publications to be able to refer to**** as well.* ***O7**** The bigger issue was that ****we actually don’t have good data****, [not so much] on the issues of anesthesia safety than on the protocols that you would need to have in place in the office-based setting for the infectious control and the potential relationship to endophthalmitis.* ***O10 So long as we have those criteria and knowledge of how to identify the “safe” from the “not as safe,”**** I think we’ll probably have a similar experience as the one we had when we went [from inpatient] to outpatient surgery. We’re all going to be nervous about it and then really happy about how well it worked out.*
*Slippery Slope*	***O7**** I know ****ophthalmologists do not want to lose access to their anesthesiologist****. They would much rather put up with having to figure out how to get them into the ASCs, having to figure out the billing issues…rather than run that risk; ****they see everything as a slippery slope****.* ***O9**** We need a major academic institution to really prove that this can be done safely…I would suggest whoever is involved in these recommendations to ****visit private ASCs and not just the best ones, but the worst ones to understand what this would look like if it was implemented everywhere.*** ***O10**** I’m afraid that CMS…or whatever ****health policy gurus look at this will be very shortsighted and say this is a savings opportunity****, rather than looking at it from a perspective of a patient benefit, patient convenience.*
*Comfort with the Status Quo*	***O3**** I think ****resistance to change type of science or psychology is going to be pretty generalizable in this situation****. The barriers to change will be proper training at the facility, proper protocols, and then surgeon acceptance.* ***O10**** …****that [operating without anesthesia care] would scare the whatever out of me****. I mean I know people are doing it, and there are a lot of people who do, there are more and more people doing it, but I’m not ready for that.* ***O11**** …people kind of say, “Oh, I’m not going to do [non-MAC sedation] because then I’d be responsible for the sedation of the patient.” Well, you’re responsible anyway.* < *laughs* > *I think some people are just comfortable having the nurse anesthetist there and ****they’ve always done it that way, so I think it’s a little bit of momentum or resistance to change.***

**Table 3:** Additional illustrative quotes regarding theme 2: financial considerations

**Financial Considerations**	
*Reimbursement Changes from Decoupling Anesthesia Care from Cataract Surgery*	***O1**** Well, the concern is that Medicare will decide to bundle the anesthesia portion of cataract surgery into the cataract surgery itself and ****reduce compensation further which they continuously do for cataract surgery now anyway.*** ***O4**** I’m suspecting that the more we go away from needing anesthesiology for cataract surgery, I think the [ophthalmologist] reimbursement will probably go down****…[payers] would say, “Oh, if they don’t need anesthesiology for this procedure, obviously, this procedure is not as difficult as it sounds.”*** ***O19**** And, you know, ****[I wonder] what would be the cost savings if we eliminated that cost of the anesthesiologist**** and so forth…*
*Lack of OBS Reimbursement*	***O13**** …we keep looking at for our practice now should we do a minor procedure and we kept looking at it. From private practice, ****the reimbursement– it doesn’t really favor having that [OBS].**** So, that’s part of it is that t****here’s not really any financial incentive to do it…****we have multiple offices and we keep talking about doing it, but we haven’t done it yet.* ***O15 …countries with nationalized health have figured out it’s less expensive to do it without layers and layers of anesthesia involved, right? …Kaiser in the United States is the same way,**** it’s the closest thing to socialized medicine we have probably. ****They do a lot of topical office-based procedures…they have incentives to lower costs and they don’t have artificial propping up of cost like we do here,**** things like physician owned ASCs…* ***O17**** …right now for example in the US we have ****penalties for doing office-based cataract surgery****. So the highest reimbursements are in the hospital-based ASC, a moderate amount in a freestanding ASC, and disincentives for doing office-based surgery.*
*Uncertain Impact on ASCs*	***O8**** I feel like, because of the lower reimbursement, cataract surgeons are being pushed to be as efficient as possible…****I can understand if some cataract surgeons might be pushing for more efficiency and wanting anesthesia not there just because of the efficiency.*** ***O10 [OBS ophthalmologists] would see an incentive in being able to do the cases when they want****, not having to be third or fourth on the totem pole below the owners in terms of scheduling cases, and if there was any financial gain to be had then they could do it… I think the ****ASC owners probably feel they have an incentive for maintaining the status quo**** because the guys that aren’t owners that come in and do cases help pay their bills.* ***O12**** Most of the people I think that are using monitored anesthesia care, they do get–**** they’re financially incentivized to use it at this point****. If you own your own surgery center or…if you’re operating out of a hospital that has anesthesia, they benefit financially from using MAC and having anesthesia as part of the surgery.*

**Table 4:** Additional illustrative quotes regarding theme 3: the interdependent relationship between ophthalmology and anesthesiology

*Interdependent Relationship Between Ophthalmology and Anesthesiology*	
*Medicolegal Risk of Assuming Sedation Roles from Anesthesia Personnel*	***O9**** I want, A, to do whatever I do as safe as possible, but B, ****if something goes sideways, I want to make sure that I’m legally also covering myself**** that, ‘Well, Doc, how in the world are you going to do chest compressions, and how are you going to mess with meds? How are you going to do all this?’* ***O14**** I think if the [American] Academy of Ophthalmology came out and said, “Hey listen, we think office-based sedation without MAC is a great approach and this is kind of the– we endorse this approach,” I think that would help encourage me to move in that direction…****because I would feel like medicolegally I have the backing****…and I’d feel more like I’m on more secure ground.* ***O15 So is it a liability thing or is it really worried about medical risk?**** Two separate questions because I don’t think that there’s any evidence that doing the eye surgery with topical anesthesia is going to increase their morbidity so I don’t think that the proximity to hospital is relevant for that reason. But you could make the argument that if this person has a five percent chance of having a heart attack today and if [it] happens during your eye surgery is that liability greater?*
*Deep Trust in Anesthesia Colleagues*	***O2**** I think for my more medically fragile patients, ****I appreciate the inputs from my anesthesia colleagues****. If there’s something they come across that they think is a red flag or something they feel like is something that we should be more cautious about, I appreciate that input.* ***O7**** I will tell you my bias, and given culture, exposure, whichever, is ****I don’t have enough experience with other [non-anesthesia] providers for me to feel that I would be comfortable with anyone other than an anesthesiologist when I really need help.*** ***O8**** I’d rather the default to be “Anesthesia is present, and I can choose specific cases to go just local” rather than the default being local, and then I have to try to find the anesthesiologist. ****When you know you already have an anesthesiologist, you’re also getting a really good workup****, like a pre-op workup.*
*Anesthesia Resource Utilization*	***O4 Because of the anesthesiology provider shortages, they’ve had to close rooms****, so I’m booking out to end of March now. Then the only way out [of scheduling delays] is going to be this nurse sedation.* ***O11 There’s a lot of just kind of hand-holding and socializing going on in the cataract clinic that really doesn’t use [anesthesiologists’] talents****, you know. So there is– I have seen absolutely no resistance by anyplace by the anesthesiologists or nurse anesthetists [moving away from routine anesthesia care for cataract surgery]…they’re just seeing it as a good transition so they can use their expertise better for people that really need them…*

*OBS* office-based cataract surgery

### Theme 1: inertia

We defined inertia as the tendency to continue established practices that serve to maintain the status quo. It manifests as a lack of impetus to change or innovate unless external pressures or compelling new evidence motivate the shift, often strengthened by a desire to avoid any potential risks that might accompany a major change in practice.

#### Lack of evidence and data

Multiple participants suggested that ophthalmologists may be reluctant to consider moving away from routine anesthesia care due to a lack of evidence justifying the change. As one said “*I’m not sure that we have the data…in terms of the patient experience or patient outcome*…” Others revealed that they were unlikely to consider changing sedation methods until they saw patient driven data that “*create[d] evidence-based guidelines.*” One participant hypothesized that, once the body of evidence supporting the safety of a move away from routine anesthesia care was present, “*a certain proportion of the population will pick up the practice just because it makes sense to them economically, operationally, and so on*.”

#### Slippery slope

Ophthalmologists worried that widespread implementation of non-anesthesia-led sedation approaches would be associated with regret if there were negative consequences to patient safety and physician access to surgery centers. “*You don’t want to have to give up something and then regret giving it up later*…” one said. Another participant detailed their experience with unforeseen ripple effects after moving to office-based surgery and no longer being able to care for patients who would clearly require anesthesia care due to their age or inability to cooperate with the surgeon intraoperatively: *“…I did so few cases [at the ASC] they took away my privileges…So now I refer them out*.” Others worried about patient harm, with one saying: “…*if you start allowing this…will there be a trail of blood? …We can’t assume that the level of responsiveness and expertise at an academic center is going to find its way into the community…”.*

#### Comfort with status quo

Multiple participants expressed a complacency and contentment with current cataract sedation norms. Even though they had prior experience with non-MAC sedation, “*it’s easier for me to just do what’s standard, what works*,” and that they just “*go with what is routine…[to] just concentrate on outcomes rather than the protocol,*”. These entrenched practices may be difficult to change, with another participant describing any alternative to anesthesia care as “*something I may not have considered before…mainly because it [MAC] was just the way that we always did it…”* Participants also wondered why they should change what’s already working: “*it just seemed like…well, that’s what we do and that’s the safe way to do it…”*, and “*there’s an anesthesiologist here or a CRNA…so…why wouldn’t we do it?*”.

### Theme 2: financial considerations

#### Reimbursement changes resulting from decoupling anesthesia care from cataract surgery

Participants worried about possible negative impacts to reimbursement that could result from decoupling anesthesia care as the default approach to cataract surgery sedation. Some worried about a possible “*over-step from the CMS,*” in other words, that the Centers for Medicare & Medicaid Services (CMS) may encroach upon physician territory to decrease reimbursement beyond acceptable norms. Arguments in favor of maintaining routine anesthesia care “*don’t have anything to do with what’s best for the patient or what’s best for that particular surgeon…it’s more of a long view on how do [ophthalmologists] dominate the market*” to maintain authority over the reimbursement schedule. Others were concerned regarding possible decreases in overall reimbursement, with one saying: “*…if the nurses are able to do the sedation for this, then I think the reimbursement for anesthesiologists automatically for this procedure will probably be lower.*”

#### Lack of OBS reimbursement

While office-based cataract surgery (OBS) is one of the most common environments where non-MAC sedation is practiced, multiple participants believed that its lack of reimbursement by Medicare was a strong external barrier to changing practice. One participant said OBS is “*either going to be self-pay or you’ve got to negotiate it with the [insurance] carriers one-on-one*.” Others expressed their desire for a sustainable system that maintains current cataract reimbursement rates even if sedation approaches were to change, with one participant claiming that ophthalmologists’ “*big fear with OBS is the moment you take away the collective bargaining power of an ASC or a hospital, you basically open up the possibility for [payers] to ratchet down how much we’re paid drastically*.”

#### Uncertain impact on ASCs

Multiple participants speculated that ophthalmologists with a financial stake in or ownership of ASCs would have a more difficult time accepting major changes to sedation techniques. For example, ASCs *“…are very expensive to set up…[and] if you have surgeons who are doing five minute cases and do 20 surgeries in a day…all this going out of there [to office-based locations], that would be financially horrible.*” Conversely, some theorized that ASC owners would be open to decoupling anesthesia care, with one participant saying: “*I can see that there potentially would be financial advantages [to moving away from anesthesia care] if [ASC owners] felt like they could operate with [a] lower level of training…in their nursing and support staff.”*

### Theme 3: interdependent relationship between ophthalmology and anesthesiology

#### Medicolegal risk of assuming sedation roles from anesthesia personnel

Apprehension existed regarding the possible medicolegal liability ophthalmologists would be taking on if they assumed more responsibility for managing patients’ sedation during surgery. One participant said: “*You know, I am very risk averse so I would want [anesthesia staff] there every day. It’s like insurance in some ways.*” Several participants articulated a clear distinction between medical and legal forms of risk. As one explained, “*Medical understanding sometimes [has] very little to do with legal liability*,” noting that practices which might intuitively appear to entail “*a lot of legal liability*” are often downplayed, whereas actions that are “*actually medically harmful*” may carry “*very little legal liability*.” Rather than not wanting to perform cataract surgery without anesthesia support, others felt that the aversion to non-MAC sedation was mostly related to the fear of retribution for poorly managing unanticipated emergent events, with one theorizing: “…*I think if someone dies on the table, whether you have MAC or not, you’re going to face a lawsuit.”*

#### Deep trust in anesthesia colleagues

Many ophthalmologists expressed deep appreciation towards the preoperative evaluation that anesthesiologists provide. One explained that it “*definitely allows me to concentrate more on the surgery itself or the things around what I feel is more comfortable for my scope of practice*,” and that at their prior practice, which did not routinely use anesthesia care, “*I would feel really uncomfortable if we didn’t have an anesthesia practitioner close by*.” Multiple others also commented positively on anesthesiologists’ intraoperative expertise: “*I really like it because I don’t have to worry about anything else that is going on with the patient…[if] the patient is uncomfortable or if I have a complication and I need some more time, then I know that the anesthesiologist can help me.”* This allows the ophthalmologist to feel “*free to take care of my surgery without having to think about anything else.”*

#### Anesthesia resource utilization

Ophthalmologists were cognizant that current cataract sedation practices may be an inefficient utilization of finite anesthesia resources. While many interview participants held sentiments like “*…it’s absolutely fantastic to have an anesthesia professional there…,”* some also conceded that “*whether or not it’s using them to the best value, I would say that’s something that could be definitely explored…*” There exists an inherent tension within the healthcare system, as “*everyone wants to think that we can just do more with less and that’s probably not the case…”* With impending relative shortages of anesthesia personnel and increasing demands for their services, ophthalmologists admitted that “*we’re going to have to be more innovative with models to deliver value-added care, and there’s going to be some tradeoffs…[we must] be thoughtful about the kind of tradeoffs we make and understanding what that means.”*

## Discussion

To the best of our knowledge, this is the first study to characterize ophthalmologists’ perceptions of the institutional barriers to changing cataract surgery sedation practices in the United States. Participants identified multiple challenges to changing current practice patterns, including financial and patient safety concerns, as well as an appreciation for the existing collaborative relationship between specialties. Many expressed some uncertainty about how losing access to routine anesthesia care might affect the future practice of cataract surgery.

Many ophthalmologists’ comments pointed to the major theme of inertia – the lack of momentum that makes it difficult to change the status quo. While some ophthalmologists were willing to consider tailoring their sedation approach according to patient need, they were reluctant to initiate or advocate for such changes on their own. This inertia appeared to be reinforced by longstanding mutually beneficial relationships between ophthalmologists and anesthesiologists, which help align financial and operational incentives within current cataract surgery care models. Implementation science studies characterize this phenomenon as the resistance to change, underscoring the challenge of implementing any clinical practices that disrupt the status quo (Rehman et al. 2021; Gupta et al. 2017; Morris et al. 2011; Balas and Boren 2000). In addition, many ophthalmologists mentioned that their colleagues who own ASC shares may have financial conflicts of interest, which makes it even more challenging to implement new sedation models for cataract surgery.

Despite these barriers, other ophthalmologists have championed alternate models of care, including performing cataract surgery in office-based surgical suites under oral or topical sedation without routine anesthesia care (Kugler et al. 2023; 2024). Our study participants agreed that vertically integrated healthcare systems, where financial incentives tend to be more aligned, would be more favorable environments for implementing non-anesthesia-led models of care (Ianchulev et al. 2016). However, for those operating within fee-for-service payment models, participants identified lower reimbursement due to the lack of dedicated facility payments for OBS as a significant barrier to broader adoption. While universal OBS may not be appropriate for all cataract surgery patients, participants expressed that making alternatives to anesthesia-led sedation more financially appealing would allow ophthalmologists to better tailor anesthetic care appropriately to higher risk patients while streamlining care and improving the patient experience for low-risk patients. (Hall et al. 2025)

Multiple participants expressed a desire for more robust evidence that alternatives to routine anesthesia care were safe and efficacious. International studies and American case series suggest the safety of relying less on routine anesthesia care (Ianchulev et al. 2016; Hamilton et al. 1988), but OBS opponents frequently cite a lack of rigorous safety data (Baker-Schena 2017). For example, most current studies are retrospective, with case series often excluding patients that required anesthesia care, which can lead to patient selection and reporting biases. A prospective, adequately powered non-inferiority trial comparing anesthesia-led versus surgeon-directed (or other non-anesthesia-led) sedation models in an appropriately selected cataract surgery population may help clarify safety, outcome, and cost implications.

Cataract surgery is estimated to account for > $3.4 billion in annual Medicare spending (Schein et al. 2012; Brown et al. 2013; Pershing et al. 2023), and ancillary costs—which include anesthesia care—comprises a significant share of the total annual cost (Pershing et al. 2023). Therefore, expanding the landscape for cataract surgery sedation to less resource-intensive approaches remains a priority. Our study participants identified several issues that need to be thoughtfully addressed to facilitate any transition to models of sedation that allocate anesthesia care according to patient risk and procedural necessity. Rather than pursuing all or none cost-containment efforts, additional evidence of feasibility and safety, innovative changes to physician reimbursement, and incentivizing incremental change could help motivate ophthalmologists to adopt physician-initiated changes to cataract surgery sedation over time (Hall et al. 2025).

### Strengths/limitations

The purpose of this study was to inductively understand ophthalmologist beliefs regarding barriers that prevent a more individualized approach to anesthesia care allocation during routine cataract surgery. This study was subject to purposive sampling, which makes it challenging to fully catalogue all ophthalmologists’ sentiments and may limit the data’s generalizability. In addition, our sample may have included a higher proportion of surgeons who do not routinely utilize anesthesia care compared with the overall ophthalmology community. However, the themes and subthemes elicited from these participants echoed the ones shared by ophthalmologists without any non-anesthesia-led sedation experience and provided a more nuanced perspective than if we had only interviewed ophthalmologists who operate with routine anesthesia care. While multiple practice settings such as HOPD, ASC, minor procedure rooms, and office-based surgery suites were represented, beliefs specific to each location were not compared due to the modest sample size. Data on participant race and ethnicity were acquired but not reported in this manuscript to minimize reidentification risk of study participants due to the relative scarcity of practicing minority ophthalmologists. Despite these limitations, we remain optimistic that this study has expanded our understanding of the current barriers to changing routine anesthesia-led sedation practices for cataract surgery.

## Conclusion

Ophthalmologists identified multiple barriers to changing current anesthesia-led sedation models for cataract surgery, including the lack of sustainable ophthalmologist reimbursement for OBS, lack of rigorous evidence of safety and feasibility of alternative sedation approaches, and ongoing inertia. Our findings can help inform future efforts to better align anesthesia care with patient and procedural needs in an aging US population.

## Supplementary Information

Additional file 1.

## Data Availability

Datasets generated and analyzed during this study are not publicly available to preserve the privacy and anonymity of study participants. Redacted versions may be available upon reasonable request.

## Abbreviations

MAC: Monitored Anesthesia Care
IV: Intravenous
US: United States
COREQ: Consolidated criteria for reporting qualitative
CFIR: Consolidated framework for implementation research
GA: General Anesthesia
ASC: Ambulatory Surgery Center
HOPD: Hospital Outpatient Department
OBS: Office Based Cataract Surgery
