# Supplementary material for: “Just the way we always did it”: ophthalmologist perspectives on changing routine anesthesia care for cataract surgery in the United States
Source: Perioper Med (Lond). 2026 May 11;15:61. doi: 10.1186/s13741-026-00697-y (PMC13330146; doi:10.1186/s13741-026-00697-y)
Supplement: Supplementary file 1 — Additional file 1. [file 13741_2026_697_MOESM1_ESM.pdf]

Additional File 1: Semi-Structured Interview Guide for Ophthalmologist Interviews

Interviewer to only read the questions, not the headings or definitions.

Introduction: The aim of the interview is to help us understand your views on monitored anesthesia care (MAC) for cataract surgery patients. There are no right or wrong answers here; we are trying to understand how different ophthalmologists approach this issue, and I'm really interested in knowing your honest opinions, both positive and negative.

Before we begin, are there any time limits that we need to be aware of?

This interview is being audio-recorded so that we can make a written transcript later for data analysis. When we transcribe this interview, we will not use your name and we will not associate anything that you say with your name. You can decline to answer any question. Your responses will only be used for the current study. The transcripts and notes will be destroyed once the data have been analyzed and reported in the aggregate.

I'll also take some notes during the interview. Do you have any questions before we begin?

(Start recording)

1) I'd like to start by asking you to tell me about your typical day in the operating room. Who is in the room with you, and what kind of cases are you doing?

2) What types of sedation do you employ in your practice?
   a. Nurse sedation
   b. Anesthesia-led sedation
   c. Have you had experience performing cataract surgery with other types of sedation models besides the one(s) you just listed?
      i. Prior training
      ii. Different practice setting

For ophthalmologists who practice with MAC:

If Anesthesia-led sedation (if not anesthesia-led, go to Question 3 on Page 4):

3) What is the role of the anesthesiologist in your current practice?
   a. What do you like about having an anesthesiologist involved in the care of your cataract surgery patients?
   b. What do you dislike about working with anesthesiologists?

4) I'm finding that people have different definitions of MAC. In your own words, what does MAC mean to you? [***Define MAC if ophthalmologist does not mention both monitoring and sedation****:* Just to get us on the same page for the rest of the interview, I'm going to spend a minute on definitions. When I say MAC, I'm talking about monitored anesthesia care, which is when an anesthesiologist or CRNA provides intraoperative monitoring and administers sedative medications during a surgical procedure. **[If they answered correctly say, "Yes, I'm glad you**

mentioned both sedation and monitoring. ***As you know…"]*** MAC is distinct from general anesthesia (GA), in which an anesthesiologist or CRNA anesthetizes a patient and secures the patient's airway by placing an endotracheal tube or laryngeal mask airway (LMA) after anesthesia induction*.* MAC is also different from the sedation that may be administered by a **sedation nurse**, which is sometimes described as "conscious sedation" or "nurse sedation." The most important difference is that anesthesia-trained providers can transition from light sedation to general anesthesia, which includes managing the airway, whereas sedation nurses cannot. Do you think this matches your personal definition of MAC? Is there anything you'd like me to clarify or explain further?

   a. With those distinctions in mind, how often do your patients receive MAC in your current practice?
      i. How often do your cataract patients receive MAC?
      ii. How often do they receive general anesthesia?
   b. How does MAC affect your ability to perform cataract surgery?
   c. In your clinical experience, how does performing surgery with MAC differ from performing surgery with the other types of sedation approaches that you use routinely?

5) Some ophthalmologists feel that MAC is necessary for all cataract surgeries. Others feel that cataract surgery can be safely performed without MAC. What are your thoughts?
   a. How do your clinical partners approach cataract surgery sedation?
   b. Do you know of any ophthalmologists who perform cataract surgery without MAC?
   c. Can you tell me about any ophthalmologists in your practice or elsewhere who currently practice with MAC but who are in favor of removing MAC from cataract surgery? What are their arguments for it?

6) What are your concerns if you were to eliminate the routine use of MAC for cataract surgery in your own practice?
   a. What are the conditions under which you might be willing to perform cataract surgery without an anesthesiologist or CRNA administering MAC intraoperatively?
      i. Sedation nurse?
      ii. Oral sedation given in preop?

7) Now, I'm going to ask you about the use of MAC in a different context to learn more about how you think about MAC for cataract surgery. For other low-risk procedures, for example, colonoscopy or cardiac catheterization, many patients are routinely scheduled for their procedure without MAC, and MAC is only requested if the proceduralist feels that the patient warrants closer monitoring.
   a. What are your thoughts if you were asked to employ this more selective application of MAC for cataract surgery?
      i. What are the steps it would take to get there?
   b. What are your major concerns about MAC no longer being the default pathway for cataract surgery?
      i. Safety/Efficiency
      ii. Finances
      iii. Impact on trainees
      iv. Preoperative evaluation
      v. Ability to perform regional blocks

8) Let’s say that the world has shifted toward this more selective application of anesthesia support for cataract surgery. When considering patient safety and surgical outcomes, what are some patient characteristics that would make you more likely to perform cataract surgery with MAC?
    a. Simple vs. complex cataract surgery
    b. How about without MAC?

9) If you or your leadership wanted to change the routine model of sedation for cataract surgery, what would that look like? Describe what steps would have to happen.
    a. How do you think others in your practice would react if there were a new protocol to eliminate MAC from routine cataract surgery? How would you react?

10) Some people who no longer routinely use MAC still find value in having an anesthesiologist onsite for emergencies. What do you think of this idea?

11) Tell me about a time when an emergent situation occurred while you were operating on a cataract surgery patient.
    a. What role, if any, did the anesthesiologist play during the emergency you described?
    b. How often do these types of adverse events or emergent situations occur at your institution? (i.e., in the last month? In the last 6 months? In the last year?)

12) What are your thoughts on anesthesia staffing?
    a. Current state
    b. Future needs

13) What are your thoughts on reimbursement for cataract surgery?
    a. How do you think this might influence the current model of sedation for cataract surgery?
    b. What are some of the financial implications of changing the sedation model for cataract surgery?
        i. For ophthalmologists?
        ii. For anesthesiologists?
        iii. For patients?

14) What strategies do you use to stay up to date with the latest evidence and practice in your specialty?
    a. Are you a member of any professional organizations at the state or national level within your specialty? What role do your professional organizations play in your clinical practice?
    b. What role would these professional organizations have, if any, in changing the approach to cataract surgery sedation?
    c. Who do you feel has significant influence on decisions related to your clinical practice?

15) Finally:
    a. What question do you wish I’d asked today?
    b. What else should I know about the role of MAC in your clinical practice?

For ophthalmologists who practice without routine MAC:

3) What is the role of the anesthesiologist in your current practice?
    a. What do you like about having an anesthesiologist involved in the care of your cataract surgery patients?
    b. What do you find challenging about working with anesthesiologists?

4) I'm finding that people have different definitions of MAC. In your own words, describe what MAC means to you. [***Define MAC if ophthalmologist does not mention both monitoring and sedation****:* Just to get us on the same page for the rest of the interview, I'm going to spend a couple of minutes on definitions. When I say MAC, I'm talking about monitored anesthesia care, which is when an anesthesiologist or CRNA provides intraoperative monitoring and administers sedative medications during a surgical procedure. **[If they answered correctly say, "Yes, I'm glad you mentioned both sedation and monitoring.** ***As you know…"]*** MAC is distinct from general anesthesia (GA), in which an anesthesiologist or CRNA anesthetizes a patient and secures the patient's airway by placing an endotracheal tube or laryngeal mask airway (LMA) after anesthesia induction*.* MAC is also different from the sedation that may be administered by a **sedation nurse**, which is sometimes described as "conscious sedation" or "nurse sedation." The most important difference is that anesthesia-trained providers can transition from light sedation to general anesthesia, which includes managing the airway, whereas sedation nurses cannot. Do you think this matches your personal definition of MAC? Is there anything you'd like me to clarify or explain further?

    a. With those distinctions in mind, how often do your patients receive MAC in your current practice?
        i. How often do your cataract patients receive MAC?
        ii. How often do they receive general anesthesia?
    b. How does MAC affect your ability to perform cataract surgery?
    c. In your experience, how does performing surgery with MAC differ from performing surgery with the other types of sedation approaches that you use routinely?

5) Right now, most ophthalmologists in US perform cataract surgery with MAC. We're interested in learning more about cataract surgeries that can safely be performed without MAC. How did you get comfortable performing cataract surgery without MAC?
    a. How do your clinical partners approach cataract surgery sedation?
    b. Do you know of any other ophthalmologists who perform cataract surgery without MAC?
        i. **[If yes:]** How do you think these ophthalmologists got comfortable performing cataract surgery without MAC?
            1. practice setting?
            2. prior training?
            3. personal characteristics?

6) For your own practice, what were your biggest concerns about eliminating the routine use of MAC for cataract surgery before you made the leap?
    a. Safety? Efficiency? Finances? Impact on trainees? Preoperative evaluation? Blocks?
    b. How did others in your practice to react when you first suggested eliminating MAC from routine cataract surgery? How did you get them on board?

7) Now, I'm going to ask you about the use of MAC in a different context to learn more about how you think about MAC for cataract surgery. For other low-risk procedures, for example, colonoscopy or cardiac catheterization, many patients are routinely scheduled for their procedure without MAC, and MAC is only requested if the proceduralist feels that the patient warrants closer monitoring.
    a. Does this more selective application of MAC (or anesthesia support) for cataract surgery accurately describe your current practice, or how is yours different?
    b. In your practice, what was the history behind getting to this stage of routinely performing cataract surgeries without MAC? Could you describe the changes that took place to make this possible?
        i. Institutional culture? Logistics? Payments? Patient expectations?
    c. What are your ongoing concerns about using this model of sedation?

8) When considering patient safety and surgical outcomes, what are some patient characteristics or clinical scenarios that would make you more likely to perform cataract surgery with MAC?
    a. Simple vs. complex cataract surgery
    b. How about without MAC?

9) Some people who no longer routinely use MAC still find value in having an anesthesiologist onsite for emergencies. What do you think of this idea?

10) Tell me about a time when an emergent situation occurred while you were operating on a cataract surgery patient.
    a. What role, if any, did the anesthesiologist play during the emergency you described?
    b. How often do these types of adverse events or emergent situations occur at your institution? (i.e., in the last month? In the last 6 months? In the last year?)
    c. What happens when an emergency arises intraoperatively in a patient who is not getting MAC?

11) Can you tell me about any ophthalmologists in your practice or elsewhere who currently practice with MAC but who are in favor of removing MAC from cataract surgery?
    a. Are they in the process of making these changes to their practice?
        i. If yes, what are some of the major challenges to transitioning their practices to be less reliant on MAC?
        ii. If no, what do you think might be preventing them from making the transition to routinely performing cataract surgery without MAC?
            1. If you had to rank these barriers from most important to least important, what would you list as your top three? How would you rank those?

12) What are your thoughts on anesthesia staffing?
    a. Current state
    b. Future needs

13) What strategies do you use to stay up to date with the latest evidence and practice in your specialty?

a. Are you a member of any professional organizations at the state or national level within your specialty? What role do your professional organizations play in your clinical practice?
b. Who do you feel has significant influence on decisions related to your clinical practice?

14) What are your thoughts on reimbursement for cataract surgery?
    a. How do you think this might influence the current model of sedation for cataract surgery?
    b. What are some of the financial implications of changing the sedation model for cataract surgery?
        i. For ophthalmologists?
        ii. For anesthesiologists?
        iii. For patients?

15) Finally:
    a. What question do you wish I'd asked today?
    b. What else should I know about the role of MAC in your clinical practice?
